# Case Report: Late-Onset Temporal Lobe Epilepsy Following Subarachnoid Hemorrhage: An Interplay Between Pre-existing Cortical Development Abnormality and Tissue Damage

**DOI:** 10.3389/fneur.2021.599130

**Published:** 2021-02-09

**Authors:** Anna Ikawa, Ayataka Fujimoto, Yoshifumi Arai, Yoshiro Otsuki, Toshiki Nozaki, Shimpei Baba, Keishiro Sato, Hideo Enoki

**Affiliations:** ^1^Comprehensive Epilepsy Center, Seirei Hamamatsu General Hospital, Hamamatsu, Japan; ^2^Department of Neurosurgery, Seirei Hamamatsu General Hospital, Hamamatsu, Japan; ^3^Department of Pathology, Seirei Hamamatsu General Hospital, Hamamatsu, Japan

**Keywords:** case report, epileptogenicity, parenchymal hemosiderosis, hippocampal atrophy, malformation of cortical development, multiple pathology

## Abstract

Epileptogenicity following brain insult depends on various factors including severity of the resulting lesion and extent of brain damage. We report a 54-year-old female patient who developed medically refractory epilepsy resulting from the interplay of pre-existing and post-insult pathologies. She presented with subarachnoid hemorrhage (SAH) due to a ruptured aneurysm and underwent clipping surgery. Seizures started 3 months post-operatively. MRI revealed cerebral ischemia and hemosiderin deposits in the left temporal lobes, and left hippocampal atrophy was suspected. As anti-seizure medications and vagus nerve stimulation failed to control her seizures, she underwent left temporal lobe resection and placement of a ventriculoperitoneal shunt for the post-operative complication of hydrocephalus. She remains seizure-free to date. Neuropathology revealed a previously undiagnosed focal cortical dysplasia (FCD) type 1a. Brain insult likely had a second hit effect in the late onset of epilepsy in this patient with pre-existing mild MCD, in whom secondary epilepsy can be attributed to the interplay of multiple underlying pathologies.

## Highlights

- Pre-existing and post-insult pathologies induced epileptogenicity
- Late-onset medically refractory epilepsy with undiagnosed FCD

## Background

Parenchymal hemosiderosis is a risk factor for focal epilepsy after intracerebral bleeding (1, 2). Epileptogenicity due to hemosiderosis is well-known and can be related to cavernous malformation (3), brain tumor (4), or intracranial hemorrhage (5, 6). The mechanism of epileptogenicity due to hemosiderosis includes abnormalities in neurotransmission and free radical formation (7).

In neuropathology of temporal lobe epilepsy, epileptogenicity has been attributed to various lesions including hippocampal sclerosis, malformation of cortical development (focal cortical dysplasia—FCD—and gray matter heterotopia), as well low-grade tumors (8, 9).

In daily practice, acute or remote damage resulting from a previous insult is considered as the sole cause of epileptogenicity. As many patients do not require surgery, information on etiology derives solely from magnetic resonance imaging (MRI). However, many pre-existing and yet undiagnosed factors as well as complications from an acute insult can impact the establishment of an epilepsy network by affecting the neurotransmitter system and intracellular and extracellular homeostasis. Indeed, it is common clinical experience that some patients develop epilepsy following an insult and others do not, even though they have the same degree of brain damage.

We have previously identified the presence of an underlying FCD as a significant factor associated with epilepsy in patients who developed post-traumatic epilepsy following severe head trauma (10). We postulated that multifactorial mechanisms might be involved in epileptogenicity following an insult, and this further applies to patients with chronic seizures following subarachnoid hemorrhage (SAH).

Here, we describe the management of a patient who developed epilepsy and who initially presented for medical care due to a ruptured aneurysmal SAH. In addition to multiple complications due to her SAH (which included acute ischemic stroke with hemorrhagic transformation, hemosiderosis and hydrocephalus), her seizures became medically refractory and warranted resection surgery, leading to the discovery of a previously unknown area suggestive of FCD type 1a.

## Case Presentation

A 54-year-old right-handed female was evaluated at the Comprehensive Epilepsy Center, Seirei Hamamatsu General Hospital (Hamamatsu, Japan) due to medically refractory weekly epileptic seizures.

Past medical history was relevant for a SAH due to a ruptured left middle cerebral artery aneurysm and status post-aneurysm clipping 11 months prior. Her clinical evolution during acute care was complicated by a hemorrhagic cerebral infarction in the left temporal lobes due to symptomatic cerebral vasospasm.

Focal aware and unaware seizures started 3 months after the surgery. Seizure semiology consisted of an epigastric sensation followed by aphasia and then loss of awareness, with occasional focal to bilateral tonic-clonic seizures. The electroencephalogram (EEG) showed interictal epileptiform discharges over the left fronto-temporal region, in keeping with topography of the bleeding and complications.

Levetiracetam, Zonisamide, Valproic acid, and Lamotrigine did not control her seizures, thus fulfilling criteria for drug-resistant seizures (11, 12). The patient was considered a candidate for surgical intervention and was offered the option of either resection surgery or vagus nerve stimulation (VNS). In view of focal aware seizures, the possibility of effective application of the magnet at the onset of seizures to abort their progression was preferred by the patient and her family. Therefore, she underwent VNS implantation at the age of 55-years and 3 months. VNS reduced her seizure intensity and frequency from weekly to monthly, and aiming at seizure freedom, the patient thereafter elected to undergo resection surgery.

**Figure 1:**
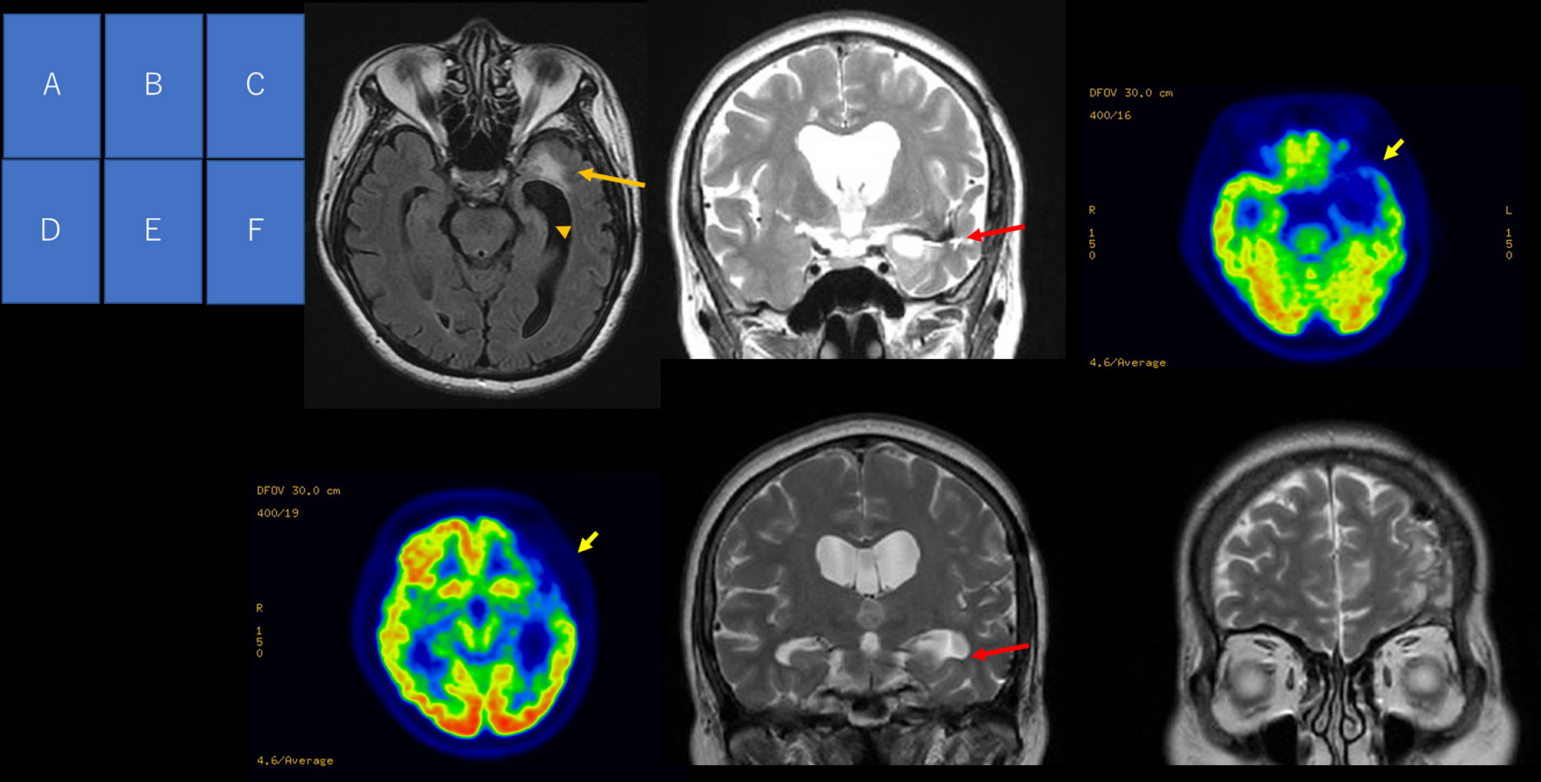
Pre-operative and post-operative neuroimaging. MRI FLAIR axial image **(A)** showing a hyperintense signal at the left temporal pole (arrow) and hippocampal atrophy (arrowhead). T2-weighted coronal image **(B)** showing a hypointense signal along the lower insula cortex and the roof of the inferior horn. Positron emission tomography **(C,D)** showing reduced glucose uptake in the left frontal and temporal lobes (arrow). T2-weighted coronal image **(E)** at the level of the hippocampal body showing hippocampal atrophy (arrow). T2-weighted coronal image at frontal region **(F)** showing a change in intensity.

Pre-surgical evaluation included MRI, 2-[^18^F]fluoro-2-deoxy-D-glucose (^18^FDG)-positron emission tomography (PET), and video EEG monitoring (VEEG). MRI showed T2 signal hyperintensity lesions in the frontal and temporal lobes, with hemosiderin deposits in the temporal area, as well as severe left hippocampal atrophy. ^18^FDG-PET showed hypometabolism in the left frontal and temporal lobe (Figure 1). VEEG captured her habitual seizures with loss of awareness preceded by an epigastric sensation and aphasia, arising electrographically from the left fronto-temporal area.

Based on the comprehensive pre-surgical evaluation, we hypothesized that her seizures were consistent with mesial temporal lobe epilepsy with a generator in the mesial temporal lobe structures from her dominant hemisphere, and we performed Spencer's anteromedial temporal *lobectomy* (a left hippocampal and amygdala resection with a temporal lobectomy from the middle temporal gyrus) (13, 14) at age 56. Surgery was complicated by subacute hydrocephalus at post-operative week 1, and she underwent ventriculoperitoneal shunting. She has remained seizure-free for more than 2-years, and remains on Levetiracetam monotherapy.

**Figure 2:**
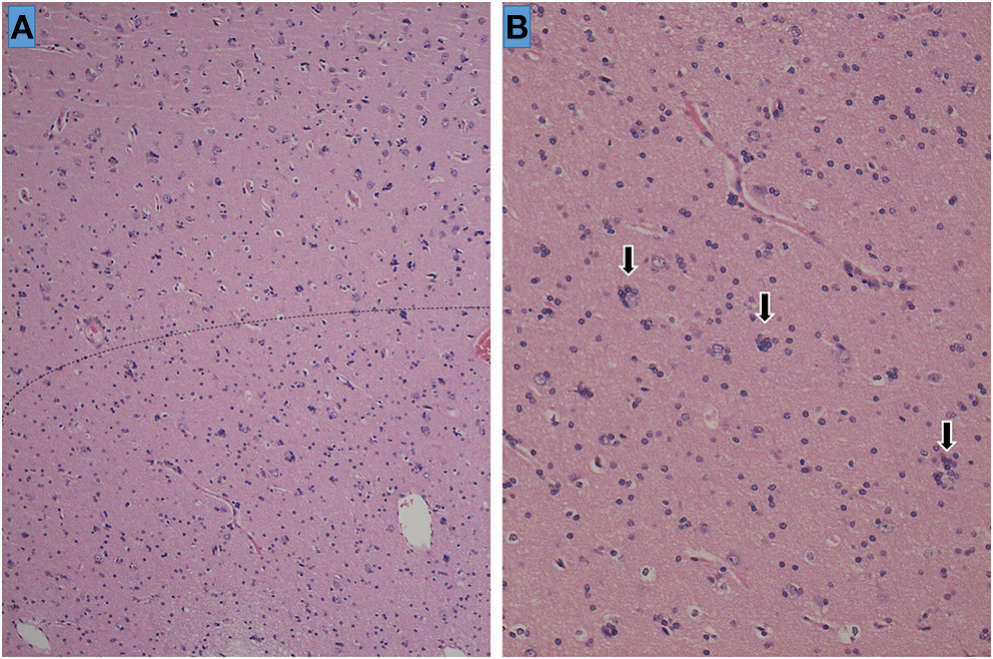
Histopathology of the left temporal lobe. The cortical-white matter border is ill-defined and indicated as a dotted line **(A)**. Sporadic ectopic neurons accompanied by glial cell proliferation (satellite oligogenesis equivalent to oligodendrocytes) surrounding the neurons (arrows) are seen at high magnification (×200) **(B)**.

Neuropathology revealed a small number of ectopic neurons in the white matter of the temporal lobe, as well as satellite oligogenesis growth, suggestive of FCD type 1a (Figure 2). Despite MRI evidence of hippocampal atrophy, the hippocampal specimen showed no neuronal loss or gliosis in CA sectors or in the dentate gyrus, and thus, no neuropathological diagnosis of hippocampal sclerosis could be confirmed.

## Discussion

In this patient, her late-onset epilepsy was at first attributed to prior SAH complicated with left frontal and temporal ischemic stroke with hemorrhagic transformation due to severe vasospasm. However, upon development of drug resistance and partial response to VNS, a full work-up for possible resection surgery, including resection of a generator in the mesial temporal structures, was postulated based on MRI signs that indicated hippocampal atrophy. Although hippocampal pathology did not confirm the presence of hippocampal sclerosis, the decision for resection surgery as the next step in this patient's management revealed FCD 1a in the temporal neocortex. This pre-existing and not yet diagnosed epileptogenic lesion could play a role in the development of epilepsy in this patient, as not all patients with a prior vascular insult will present with chronic unprovoked seizures.

Many patients, like ours, will present with recurrent seizures that develop following the diagnosis of an insult, such as stroke, head trauma, or tumors (15–21). The current use of terms such as “post-stroke epilepsy,” “post-traumatic epilepsy,” and “brain tumor-related epilepsy” can guide our management decisions and allows us to provide patients and families an overview of the predicted outcome. However, the interplay with other underlying known and unknown neuropathological factors might lead to unexpected directions, and attention needs to be paid to patients with rather poor clinical evolution.

An imbalance between excitatory and inhibitory neurotransmission causing epileptic seizures (22–24) is a common mechanism in the various etiologies of epilepsy. It is however possible that even in the presence of a highly epileptogenic brain lesion such as FCD, epileptogenicity and clinically manifested seizures in some patients only occur after a second hit/insult. Indeed, the “two-hit theory” (25) or “multiple-hit theory” (26) may explain this situation (27, 28). In our patient, seizures became controlled after resection surgery, including resection of the cortical malformation and of areas bearing hemosiderin deposits.

## Data Availability Statement

The raw data supporting the conclusions of this article will be made available by the authors, without undue reservation.

## Ethics Statement

The studies involving human participants were reviewed and approved by Submission of this case report was approved by the ethics review board at Seirei Hamamatsu General Hospital, and written informed consent was obtained from the patient. The patients/participants provided their written informed consent to participate in this study. Written informed consent was obtained from the individual(s) for the publication of any potentially identifiable images or data included in this article.

## Author Contributions

All authors made substantial contributions to the conception, validation, design, acquisition of data, or analysis and interpretation of data.

## Conflict of Interest

The authors declare that the research was conducted in the absence of any commercial or financial relationships that could be construed as a potential conflict of interest.

## Abbreviations

EEG: electroencephalogram
^18^FDG: 2-[^18^F]fluoro-2-deoxy-D-glucose
MCD: malformation of cortical development
MRI: magnetic resonance imaging
PET: positron emission tomography
SAH: subarachnoid hemorrhage
VEEG: video electroencephalogram
VNS: vagus nerve stimulation.

## References

[1] Hammen T Romstöck J Dörfler A Kerling F Buchfelder M Stefan H. Prediction of postoperative outcome with special respect to removal of hemosiderin fringe: a study in patients with cavernous haemangiomas associated with symptomatic epilepsy. Seizure. (2007) 16:248–53. 10.1016/j.seizure.2007.01.001 17276092

[2] Hirano T Enatsu R Iihoshi S Mikami T Honma T Ohnishi H. Effects of hemosiderosis on epilepsy following subarachnoid hemorrhage. Neurol Med Chir. (2019) 59:27–32. 10.2176/nmc.oa.2018-0125 30568071 PMC6350000

[3] Schuss P Marx J Borger V Brandecker S Güresir Á Hadjiathanasiou A. Cavernoma-related epilepsy in cavernous malformations located within the temporal lobe: surgical management and seizure outcome. Neurosurg Focus. (2020) 48:E6. 10.3171/2020.1.FOCUS19920 32234980

[4] Roelcke U Boxheimer L Fathi AR Schwyzer L Ortega M Berberat J. Cortical hemosiderin is associated with seizures in patients with newly diagnosed malignant brain tumors. J Neurooncol. (2013) 115:463–8. 10.1007/s11060-013-1247-7 24045969

[5] Lahti AM Saloheimo P Huhtakangas J Salminen H Juvela S Bode MK. Poststroke epilepsy in long-term survivors of primary intracerebral hemorrhage. Neurology. (2017) 88:2169–75. 10.1212/WNL.0000000000004009 28476758

[6] Räty S Sallinen H Virtanen P Haapaniemi E Wu TY Putaala J. Occipital intracerebral hemorrhage-clinical characteristics, outcome, and post-ICH epilepsy. Acta Neurol Scand. (2020). 10.1111/ane.13303. [Epub ahead of print]. 32602110

[7] Kraemer DL Awad IA. Vascular malformations and epilepsy: clinical considerations and basic mechanisms. Epilepsia. (1994) 35(Suppl. 6):S30–43. 10.1111/j.1528-1157.1994.tb05987.x 8206013

[8] Li LM Cendes F Andermann F Watson C Fish DR Cook MJ. Surgical outcome in patients with epilepsy and dual pathology. Brain. (1999) 122(Pt. 5):799–805. 10.1093/brain/122.5.799 10355666

[9] Salanova V Markand O Worth R. Temporal lobe epilepsy: analysis of patients with dual pathology. Acta Neurol Scand. (2004) 109:126–31. 10.1034/j.1600-0404.2003.00183.x 14705975

[10] Sakakura K Fujimoto A Arai Y Ichikawa N Sato K Baba S. Posttraumatic epilepsy may be a state in which underlying epileptogenicity involves focal cortical dysplasia. Epilepsy Behav. (2020) 114:107352. 10.1016/j.yebeh.2020.107352 32843304

[11] Kwan P Brodie MJ Early identification of refractory epilepsy. N Engl J Med. (2000) 342:314–9. 10.1056/NEJM200002033420503 10660394

[12] Berg AT Vickrey BG Testa FM Levy SR Shinnar S DiMario F. How long does it take for epilepsy to become intractable? A prospective investigation. Ann Neurol. (2006) 60:73–9. 10.1002/ana.20852 16685695

[13] Spencer DD Spencer SS Mattson RH Williamson PD Novelly RA. Access to the posterior medial temporal lobe structures in the surgical treatment of temporal lobe epilepsy. Neurosurgery. (1984) 15:667–71. 10.1227/00006123-198411000-00005 6504282

[14] Al-Otaibi F Baeesa SS Parrent AG Girvin JP Steven D. Surgical techniques for the treatment of temporal lobe epilepsy. Epilepsy Res Treat. (2012) 2012:374848. 10.1155/2012/374848 22957228 PMC3420380

[15] Olivecrona M Zetterlund B Rodling-Wahlström M Naredi S Koskinen LO. Absence of electroencephalographic seizure activity in patients treated for head injury with an intracranial pressure-targeted therapy. J Neurosurg. (2009) 110:300–5. 10.3171/2008.4.17538 18759609

[16] Kim HJ Lee SA Kim HW Kim SJ Jeon SB Koo YS. The timelines of MRI findings related to outcomes in adult patients with new-onset refractory status epilepticus. Epilepsia. (2020) 61:1735–48. 10.1111/epi.16620 32715470

[17] Severino M Geraldo AF Utz N Tortora D Pogledic I Klonowski W. Definitions and classification of malformations of cortical development: practical guidelines. Brain. (2020) 143:2874–94. 10.1093/brain/awaa174 32779696 PMC7586092

[18] Sales F Chaves J McMurray R Loureiro R Fernandes H Villanueva V. Eslicarbazepine acetate in post-stroke epilepsy: clinical practice evidence from Euro-Esli. Acta Neurol Scand. (2020) 142:563–73. 10.1111/ane.13323 32691850 PMC7754143

[19] Siig Hausted H Nielsen JF Odgaard L. Epilepsy after severe traumatic brain injury: frequency and injury severity. Brain Inj. (2020) 34:889–94. 10.1080/02699052.2020.1763467 32506958

[20] Uluduz D Midi I Duman T Yayla V Karahan AY Afsar N. Epileptic seizures in cerebral venous sinus thrombosis: subgroup analysis of VENOST study. Seizure. (2020) 78:113–7. 10.1016/j.seizure.2020.02.017 32353818

[21] Ersoy TF Ridwan S Grote A Coras R Simon M. Early postoperative seizures (EPS) in patients undergoing brain tumour surgery. Sci Rep. (2020) 10:13674. 10.1038/s41598-020-70754-z 32792594 PMC7426810

[22] Lotan E Tomer O Tavor I Blatt I Goldberg-Stern H Hoffmann C. Widespread cortical dyslamination in epilepsy patients with malformations of cortical development. Neuroradiology. (2020). 10.1007/s00234-020-02561-2. [Epub ahead of print]. 32975591

[23] Sarnat HB Hader W Flores-Sarnat L Bello-Espinosa L. Synaptic plexi of U-fibre layer beneath focal cortical dysplasias: role in epileptic networks. Clin Neuropathol. (2018) 37:262–76. 10.5414/NP301103 30232955

[24] Brenet A Hassan-Abdi R Somkhit J Yanicostas C Soussi-Yanicostas N. Defective excitatory/inhibitory synaptic balance and increased neuron apoptosis in a zebrafish model of dravet syndrome. Cells. (2019) 8:1199. 10.3390/cells8101199 31590334 PMC6829503

[25] Hamelin S Depaulis A. Revisiting hippocampal sclerosis in mesial temporal lobe epilepsy according to the “two-hit” hypothesis. Rev Neurol. (2015) 171:227–35. 10.1016/j.neurol.2015.01.560 25748332

[26] Galanopoulou AS. Basic mechanisms of catastrophic epilepsy – overview from animal models. Brain Dev. (2013) 35:748–56. 10.1016/j.braindev.2012.12.005 23312951 PMC3644363

[27] Chang YC Huang CC Huang SC. Long-term neuroplasticity effects of febrile seizures in the developing brain. Chang Gung Med J. (2008) 31:125–35. 18567412

[28] Colciaghi F Finardi A Frasca A Balosso S Nobili P Carriero G. Status epilepticus-induced pathologic plasticity in a rat model of focal cortical dysplasia. Brain. (2011) 134(Pt. 10):2828–2843. 10.1093/brain/awr045 21482549

